# A Double-Hit Model of Early-Life Stress and High-Fat Feeding Reveals Enhanced Exocrine Pancreatic Remodeling

**DOI:** 10.3390/biology15151297

**Published:** 2026-08-05

**Authors:** Laura García-Orozco, Carlos Alberto Mandarim-de-Lacerda, Bélgica Vásquez

**Affiliations:** 1 Doctoral Program in Morphological Sciences, Faculty of Medicine, Universidad de La Frontera, Temuco 4780000, Chile; l.garcia05@ufromail.cl; 2 Health Knowledge Management Research Group, Medical Science Faculty, Corporación Universitaria Empresarial Alexander Von Humboldt (UNIHUMBOLDT), Armenia 630004, Quindío, Colombia; 3 Laboratory of Morphometry, Metabolism, and Cardiovascular Disease, Institute of Biology, Biomedical Center, The University of the State of Rio de Janeiro, Rio de Janeiro 20551-030, Brazil; mandarim@uerj.br; 4 Center of Excellence in Morphological and Surgical Studies, Universidad de La Frontera, Temuco 4811230, Chile; 5 Department of Basic Sciences, Faculty of Medicine, Universidad de La Frontera, Avenida Francisco Salazar 01145, Temuco 4811230, Chile

**Keywords:** maternal separation, high-fat diet, exocrine pancreas, early-life stress, developmental programming

## Abstract

Adverse childhood experiences are associated with long-term metabolic disturbances, yet their effects on the exocrine pancreas remain poorly understood. Using maternal separation (MS) as a model of early-life stress, this study examined whether MS modifies the exocrine pancreatic response to a post-weaning high-fat diet (HFD) in male C57BL/6 mice. Animals were exposed to MS or standard rearing conditions and fed a control diet or HFD for 16 weeks. Body and pancreatic mass, plasma amylase and lipase activities, exocrine pancreatic histopathology, and apoptosis were assessed. HFD increased body mass and reduced the pancreas-to-body mass ratio independently of MS, without altering absolute pancreatic mass. Lipase activity increased significantly in response to both MS and HFD, whereas amylase showed a non-significant upward trend. Histopathological analysis revealed condition-dependent exocrine pancreatic remodeling, including acinar hypertrophy, autophagic vacuolization, focal necrosis, vascular alterations, interstitial edema, and ductal changes. These alterations were mild in HFD-only mice but more pronounced in MS-exposed animals, particularly when combined with HFD. Apoptosis was strongly influenced by early-life stress and modulated by diet, with significant effects of MS and the MS × diet interaction. Overall, early-life stress induces persistent structural and functional alterations in the exocrine pancreas, increasing susceptibility to nutritional challenges in adulthood.

## Simple Summary

Adverse childhood experiences have lasting health effects and increase the risk of metabolic disorders in adulthood. However, their impact on the exocrine pancreas, which is responsible for digestion, remains poorly understood. This study assessed whether early stress increases the exocrine pancreas susceptibility to high-fat diet-induced effects in adulthood. To do this, mice exposed to early maternal separation were compared with normally reared mice that were subsequently fed either a control or a high-fat diet. Childhood stress was associated with structural alterations in pancreatic tissue and changes in digestive enzyme activity. These alterations were more pronounced when early stress was combined with a high-fat diet, suggesting greater pancreatic vulnerability to later nutritional challenges. These findings highlight the importance of protecting early life conditions to prevent digestive and metabolic diseases in adulthood.

## 1. Introduction

Adverse childhood experiences (ACEs) are a critical determinant of health in later life. Early exposure to stressful events, especially during sensitive developmental periods, can induce persistent alterations in neuroendocrine, immunological, and metabolic systems and is associated with an increased risk of metabolic disorders, including obesity, insulin resistance, and impaired glucose homeostasis [1,2,3,4].

Maternal separation (MS) in rodents has become an established experimental paradigm for modeling early stress associated with ACEs [5,6,7]. This model is characterized by repeated, prolonged exposure to maternal absence during the early postnatal period, making it a chronic stressor capable of inducing lasting alterations in the regulation of the hypothalamic–pituitary–adrenal axis, behavior, and metabolism [8,9]. Additionally, studies have proposed that subsequent exposure to metabolically unfavorable environments, such as high-fat diets (HFD), may modulate the effects of early stress [10,11,12]. This approach, often described as a “double whammy” model, suggests that adverse programming induced in early life stages can interact with later nutritional challenges, exacerbating metabolic dysfunction [13].

In the pancreatic context, most studies have focused on the endocrine compartment, describing alterations in insulin secretion, glucose homeostasis, and insulin sensitivity following exposure to MS and HFD [11,14,15]. This emphasis has influenced the interpretation of the effects of early stress on the pancreas, prioritizing the analysis of the endocrine compartment and neglecting the evaluation of possible specific responses from the exocrine compartment [16]. In this regard, evidence from studies of pancreatic development and endocrine-exocrine communication indicates that both compartments exhibit partially distinct differentiation trajectories, regulatory mechanisms, and critical windows of susceptibility [17,18], supporting the plausibility of divergent responses to the same adverse stimulus.

In parallel, the available evidence on the exocrine pancreas primarily comes from independently evaluated models, in which stress or metabolic overload is analyzed in isolation. Within this framework, chronic immobilization stress models in rats have demonstrated significant increases in serum amylase and lipase levels, accompanied by pancreatic structural damage characterized by acinar cell destruction, cytoplasmic vacuolization, inflammatory cell infiltration, interacinar and interlobular fibrosis, ductal dilation, and adipose tissue accumulation [19,20]. Similarly, HFD has been reported to induce alterations in the exocrine pancreas, including pancreatic fat accumulation, fibrosis, acinar damage, and changes in serum levels of exocrine pancreatic enzymes [21]. However, these findings do not account for the interaction between early programming and subsequent dietary exposure.

From a translational perspective, the identified gap is particularly relevant, as the exocrine pancreas plays a central role in clinically relevant disorders such as pancreatitis, pancreatic steatosis, and digestive alterations associated with obesity [22]. These conditions could be influenced by early exposure to stress and Western diets. Experimental models that integrate early stress with subsequent nutritional challenges provide a suitable framework to explore whether early adverse experiences program a heightened structural vulnerability of the exocrine pancreas to later metabolic insults.

Based on this framework, we hypothesized that MS does not merely exert an independent effect on the exocrine pancreas but rather modulates the morphological response to a post-weaning HFD, resulting in additive or interactive effects on acinar architecture and tissue organization. Accordingly, the present study aimed to evaluate the effect of MS, in combination with a post-weaning HFD, on morphological parameters of the exocrine pancreas in a murine model, as an initial approach to identify structural alterations that may underlie increased susceptibility to metabolic stress in adulthood.

## 2. Materials and Methods

### 2.1. Animals

This study used pancreatic tissue samples obtained from an experimental model developed within the UFRO-DIUFRO project (code DI24-0029). In this model, the researchers evaluated the effects of MS and a post-weaning HFD on the structure and function of the endocrine pancreas in male C57BL/6 mice. Using the same animals allowed us to extend the analysis to other metabolically relevant tissues, avoiding the use of new animals and in accordance with the 3Rs principles of reduction and refinement [23].

All experimental procedures were performed in accordance with the Guide for the Care and Use of Laboratory Animals [24]. The Scientific Ethics Committee of the University of La Frontera approved the original protocol (File Number 067_24) on 28 May 2024, authorizing the collection and analysis of exocrine pancreatic tissue and blood plasma samples. The Scientific Ethics Committee of the University of La Frontera subsequently evaluated and approved the specific analysis of these samples (File Number 006_25) on 24 January 2025.

To provide context for the experimental design and the conditions under which the experimental procedures generated the analyzed samples, this section describes the general characteristics of the protocol, including the origin and handling of the animals.

Twenty female and twenty male mice aged 6 to 8 weeks of the C57BL/6 strain (*Mus musculus*) were obtained from the Chilean Institute of Public Health. Before the start of the experiment, the animals underwent a 2-week acclimatization period at the Center of Excellence in Morphological and Surgical Studies (CEMyQ) at the University of La Frontera. Throughout the experimental phase, they were kept under controlled temperature and humidity conditions, with a 12:12 h light–dark cycle and with *ad libitum* access to food and water. For breeding, sexually mature virgin females were mated overnight with males, mating confirmed by observation of vaginal plugs; subsequently, pregnant females were housed individually. During gestation and lactation, the females received a control diet (CD) for rodents, formulated according to the AIN-93G recommendation of the American Institute of Nutrition [25]. They had *ad libitum* access to water (Table 1). The diets were prepared by PRAGSOLUÇÕES Biociências (Jaú, São Paulo, Brazil, www.pragsolucoes.com.br, accessed on 15 April 2026) and stored at −20 °C throughout the study for optimal preservation.

Seven days before birth, the researchers checked for the presence of pups by observing the dams twice daily, at the beginning and end of the light period. The researchers recorded the day of birth as postnatal day (PND) 0. On PND 2, the researchers determined pup sex by visual examination of the perianal region, following validated methods for pigmented mice [26]. In this strain, males display a pigmentation spot visible to the naked eye in the scrotal area from the first days of life. Neonatal females lack this pigmentation, enabling early and reliable sex differentiation. The litters were randomly reduced to six pups to ensure adequate, standardized nutrition during lactation. For this adjustment, each pup was individually identified, and a sequence of random numbers was generated using the RAND function in Microsoft Excel^®^ (version 16.81). After sorting these values, the six pups with the lowest numbers were selected.

### 2.2. Maternal Separation

The MS protocol followed the model previously established and validated by George et al. (2010) [27], known as “Maternal Separation with Early Weaning,” which combines prolonged periods of MS with early weaning. The experimental design included two groups: an unmanipulated group (UM), consisting of ten mothers with their respective offspring who remained together without experimental intervention throughout the pre-weaning period, with weaning performed conventionally on PND 21, and an MS group, consisting of ten mothers with their offspring, exposed to daily separation periods of 240 min between PNDs 2 and 5 and 480 min between PNDs 6 and 16, combined with early weaning on PND 17.

During the separation periods, the pups remained in their original cages, while the mothers moved to new cages in an adjacent room, where identical conditions of temperature, humidity, noise, cleanliness, and ad libitum access to food and water were maintained. The pups stayed with their littermates throughout the separation procedure, and the researchers placed the cages on a heating pad to maintain a constant temperature (32–34 °C) and support thermoregulation. After the separation period, the researchers returned the mothers to their original cages as appropriate. The researchers performed this procedure either between 9:00 a.m. and 1:00 p.m. or at 5:00 p.m., depending on the corresponding PND. The researchers carried out daily inspections throughout the procedure to detect signs of dehydration or distress, in accordance with the “Animal Monitoring” protocol described by Morton and Griffiths [28].

### 2.3. Post-Weaning Diet

At weaning, one male offspring per litter was randomly selected, and animals from independent litters were assigned to a single experimental condition. To avoid pseudoreplication, only one male per litter was included in each group; therefore, the litter was considered the experimental unit for all analyses. These animals were then allocated to dietary subgroups according to the assigned post-weaning diet (CD or HFD). The animals received the assigned diets for 16 weeks (Figure 1). This design resulted in four experimental subgroups: UM-CD and MS-CD, which received a CD providing 17.0% of total energy from soybean oil (16.53 kJ/g), and UM-HFD and MS-HFD, which received a HFD providing 32.0% of total energy from lard and 17.0% from soybean oil (20.72 kJ/g), with a sample size of five animals per subgroup and a total of 20 animals for the entire experiment. The study included only male mice because the estrous cycle can influence behavioral variables, such as anxiety and food intake [29].

The inclusion of five animals per group reflects statistical and methodological criteria commonly accepted in morphological research. This decision is consistent with the bioethical principles of the 3Rs (replacement, reduction, and refinement) formulated by Russell & Burch (1959) [23] for animal experimentation. From a statistical standpoint, Cruz-Orive & Weibel (1990) [30] indicate that a sample size of five individuals per group constitutes an appropriate starting point. In the presence of a uniform trend, the probability that the observed result is attributable to chance is *p* = (1/2)^5^ = 0.03125, which suggests the finding is potentially conclusive in the context of exploratory studies.

Due to the nature of the MS protocol and dietary interventions, it was not possible to conceal the assignment or perform blinding during the experiment, as these procedures required specific animal handling for each group.

No formal study protocol was registered prior to the initiation of the study. The research question, experimental design, and analysis plan were defined a priori.

### 2.4. Euthanasia

At the end of the 16-week dietary intervention after weaning, the mice fasted for 6 h. The researchers recorded the body mass of each animal before euthanasia and administered an overdose of ketamine/xylazine (240/30 mg/kg) to perform the procedure [31]. After confirming deep anesthesia, the researchers collected blood samples by cardiac puncture of the left ventricle and transferred them to heparinized tubes for analysis. The researchers then dissected the pancreas, measured its mass using an analytical balance (A&D Orion^®^ HR 120, A&D Technology, Saitama, Japan), and fixed the tissue for 48 h at room temperature (RT) in 4% buffered formalin (1.27 mol/L formaldehyde in 0.1 M phosphate buffer, pH 7.2; Sigma-Aldrich, St. Louis, MO, USA).

### 2.5. Plasma Analysis

Blood samples were collected in heparinized tubes and centrifuged at 3000 rpm for 15 min to separate the plasma. Plasma aliquots were prepared in sterile microtubes to prevent repeated freeze–thaw cycles and stored at −80 °C until biochemical analysis. Before analysis, samples were thawed at RT and gently homogenized. Samples with visible contamination or precipitation were excluded from the analysis.

Commercial kinetic colorimetric assays were used to determine the plasma enzymatic activity of amylase and lipase. The assays were based on the hydrolysis of chromogenic substrates, and enzymatic activity was monitored kinetically by photometric measurement of absorbance changes. Results were expressed as units per liter (U/L).

Biochemical analyses were performed using an automatic clinical chemistry analyzer (Wiener CM250, Wiener Lab Group, Rosario, Argentina). Commercial diagnostic kits came from Human Diagnostics Worldwide (Wiesbaden, Germany). Before sample processing, calibration, and internal quality control procedures were followed according to the manufacturer’s recommendations. Whenever possible, identical analytical conditions were applied to all samples, calibrators, and controls to minimize inter-assay variability.

### 2.6. Histopathological Evaluation

After fixation, the samples were dehydrated and embedded in Paraplast Plus (Sigma-Aldrich, St. Louis, MO, USA). A rotary microtome (Leica Biosystems RM 2255, Deer Park, IL, USA) produced 5 µm-thick histological sections. For histopathological evaluation, each paraffin block was sectioned at 150 µm intervals until a total of 10 sections per block were obtained, and the sections were stained with hematoxylin and eosin (H&E). These preparations supported a semi-quantitative histopathological analysis of the exocrine pancreas across all experimental groups. To address this objective, the study applied a semi-quantitative scale adapted from previously described criteria for evaluating pancreatic injury in murine models [32] and complemented with additional histopathological features reported in the literature [33]. Although studies have mainly applied this scale in the context of acute pancreatitis, its variables encompass fundamental histopathological processes in pancreatic tissue, including acinar alterations, vascular changes, interstitial and ductal involvement, and necrosis and atrophy. These characteristics enable its use in other experimental contexts to evaluate structural and tissue-level changes. Independent, blinded evaluation of the histological slides occurred without knowledge of the experimental group assignment. The analysis used an ordinal scale ranging from 0 to 3, where 0 indicated the absence of histological alterations and 1, 2, and 3 denoted mild, moderate, and severe changes, respectively. The evaluation covered eight histopathological variables: acinar hypertrophy, acinar autophagy, acinar necrosis, vascular injury, interstitial edema, inflammation and hemorrhage, fat necrosis, ductal alterations, and acinar atrophy. The analysis excluded acinar apoptosis from the semi-quantitative histopathological assessment because a specific TUNEL assay was used to evaluate it.

The analysis was performed independently by two evaluators (LGO and BV). To ensure the impartiality and reproducibility of the histopathological evaluation, all histological sections were previously anonymized and randomly coded by an independent researcher not involved in the analysis, thereby keeping the evaluators blinded to the experimental assignment throughout the evaluation process. Any discrepancies were resolved through discussion until consensus was reached. Before the analysis, a three-stage interobserver calibration process was conducted, including theoretical training, practical laboratory training, and agreement assessment. Inter-rater agreement was assessed using the weighted kappa coefficient, yielding κ = 0.83. Table 2 summarizes the operational definitions of each variable and the criteria used to assign scores.

We selected representative images from systematically and randomly sampled microscopic fields from each animal. These fields primarily represented the exocrine pancreatic parenchyma and avoided over-representation of large ducts, blood vessels, and pancreatic islets. When vascular changes or periductal inflammatory alterations were consistently observed across samples within a given experimental group, we also included images of these features to reflect the predominant histological pattern. Image selection was performed blinded to the experimental group.

### 2.7. Terminal Deoxynucleotidyl Transferase-Mediated Deoxyuridine Triphosphate (dUTP) Nick-End Labeling (TUNEL) Assay

The study assessed cellular apoptosis in paraffin-embedded pancreatic tissue samples using the TUNEL assay with a commercial kit (ab206386, Abcam, Cambridge, UK) according to the manufacturer’s recommendations. After obtaining histological sections with a thickness of 5 µm, the protocol included deparaffinization in xylene and rehydration through a graded series of decreasing ethanol concentrations (Merck, Darmstadt, Germany), followed by washing with 1× TBS (Merck, Darmstadt, Germany). For permeabilization, the sections were incubated with proteinase K (1 µL proteinase K plus 99 µL deionized water per sample) for 20 min at RT. The protocol then blocked endogenous peroxidase activity by treating the sections with 3% H_2_O_2_ in methanol (10 µL of 30% H_2_O_2_ plus 90 µL methanol; Merck, Darmstadt, Germany) for 20 min at RT.

For equilibration, the protocol applied the TdT equilibration buffer for 10 min at RT. Immediately before use, the labeling reaction mixture was combined with 1 µL of TdT enzyme with 39 µL of TdT labeling reaction mix per sample, followed by incubation of the sections for 5 min at 37 °C. For signal detection, the procedure incubated the sections with Blocking Buffer for 10 min at RT and then with Streptavidin–HRP (25×) diluted in Blocking Buffer (4 µL conjugate plus 96 µL buffer per sample) for 30 min at RT in a humidified chamber. Signal development employed DAB solution (DAB solution 1 plus DAB solution 2; 1:30 dilution) for 25 min. Finally, the protocol counterstained the sections with methyl green for 1 min, dehydrated them in 100% ethanol, cleared them in xylene, and mounted them with Entellan^®^ (Merck, Darmstadt, Germany).

For the positive control, the protocol treated the sections with DNase I (1 µg/µL) in TBS containing 1 mM MgSO_4_ (Vivantis Technologies, Shah Alam, Selangor, Malaysia) for 20 min at RT immediately after proteinase K treatment. For the negative control, the procedure omitted the TdT enzyme from the reaction mixture.

For quantitative analysis, images were acquired using bright-field microscopy with an Olympus BX53 microscope equipped with a 40× objective. Serial pancreatic sections were collected at 150 µm intervals to minimize spatial overlap between sampled areas. Twenty microscopic fields were analyzed per animal (100 fields per experimental group). Fields were selected using systematic random sampling and were restricted to the exocrine parenchyma, avoiding large ducts, blood vessels, and pancreatic islets. The field area at 40× was calibrated in Image-Pro Premier 9.3 (Media Cybernetics, Warrendale, PA, USA) and corresponded to 0.06568 mm^2^. Apoptosis was quantified as the number of TUNEL-positive nuclei per field and expressed as cells/mm^2^. Measurements were averaged for each animal prior to statistical analysis [34]. Because only one male offspring per litter was included in the study, each animal represented an independent litter, and the litter was the experimental unit for all analyses.

This study was exploratory in nature, and no single primary outcome measure was prespecified for formal sample size determination.

### 2.8. Statistical Analysis

Data were tested for normality (using the Shapiro–Wilk test for small samples) and homogeneity of variances (using Levene’s test) before being presented as means and standard deviations. Differences between groups were assessed using a two-way analysis of variance (ANOVA) and Tukey’s multiple comparisons test. Because only one male offspring per litter was included, each observation corresponded to an independent litter, which was therefore considered the experimental unit for all analyses. In all cases, a significance level of *p* < 0.05 was adopted (GraphPad Prism version 10.6.1 for Windows, GraphPad Software, Boston, MA, USA). All datasets met the assumptions for parametric analysis. Data analysis was performed using coded datasets, and group identity was revealed only after completion of the statistical analyses.

Inclusion and exclusion criteria were prespecified. Only one male offspring per litter was included to avoid pseudoreplication, and the litter was defined as the experimental unit. No animals or experimental units were excluded after allocation, as all animals completed the MS and dietary intervention protocols and were included in the final analysis. Plasma samples with visible contamination or precipitation were excluded from biochemical analyses according to predefined quality-control criteria. No additional data points were excluded beyond these criteria.

## 3. Results

### 3.1. A High-Fat Diet Increases Body Mass Without Altering Pancreatic Mass, Resulting in a Reduced Pancreas-to-Mouse Mass Ratio Independent of Maternal Separation

Analysis of body mass revealed a significant main effect of diet, with HFD-fed mice exhibiting a marked increase in body weight compared with CD-fed mice, independent of MS (Figure 2E). Specifically, body mass increased by 40.6% in UM-HFD mice relative to UM-CD mice and by 35.7% in MS-HFD mice compared with MS-CD mice. No significant effect of MS and no interaction between MS and diet were detected for this parameter (Table 3). In contrast, absolute pancreatic mass did not differ significantly among experimental groups, indicating that neither HFD consumption nor MS altered pancreatic mass at the macroscopic level (Figure 2F).

In light of these findings, the pancreas-to-mouse mass ratio was evaluated. The UM-CD group had the highest pancreas-to-mouse mass ratio, which served as the baseline for the study. A decrease was observed in the other groups, with the lowest pancreas-to-mouse mass ratio in the UM-HFD group (−28.6%), followed by the MS-HFD group (−25.3%) and the MS-CD group (−2.2%) compared to the UM-CD group. Comparisons between groups exposed to MS did not reveal significant differences in the pancreas-to-mouse mass ratio, with comparable values maintained between UM-CD and MS-CD, as well as between UM-HFD and MS-HFD (Figure 2G). In this regard, a two-way ANOVA indicated that MS had no significant effect on this parameter, nor did a significant interaction between MS and diet occur. In contrast, diet explained most of the total variability in the pancreas-to-mouse mass ratio (61.53% of the total variance), with HFD consumption driving a significant reduction regardless of MS status (Table 3).

### 3.2. Maternal Separation and Diet Differentially Modulate the Plasma Activity of Pancreatic Digestive Enzymes

Plasma activity of pancreatic amylase showed an increasing trend in the MS groups compared to the UM groups, with the highest value observed in the MS-HFD group, followed by MS-CD (Figure 3A). However, these differences did not reach statistical significance. Two-way ANOVA did not reveal a significant effect of MS or diet, nor a significant interaction between these factors for this parameter (Table 3). Despite the lack of statistical significance, the data showed a descriptive trend toward increased plasma amylase activity associated with MS.

Plasma activity of pancreatic lipase showed a trend similar to that observed for amylase, with higher values in the MS groups; however, unlike amylase, the MS-HFD group showed a significant increase, reaching a 26.5% rise compared to the UM-CD group, while the UM-HFD group showed a more moderate increase (9.2%) compared to baseline (Figure 3B). Statistical analysis revealed significant effects of both MS and diet on plasma lipase activity, with no significant interaction detected between the two factors (Table 3).

### 3.3. Maternal Separation and a High-Fat Diet Promote Structural Alterations of the Exocrine Pancreas, with Predominant Involvement of Acinar Cells

Histopathological evaluation of the exocrine pancreas revealed a lesion profile dependent on the experimental condition. The UM-CD group showed no histological alterations in all evaluated categories, with acini exhibiting preserved morphology and tissue architecture, thus constituting the baseline pattern of the study (Figure 4). In this group, the pancreatic acini appeared compact and uniformly organized, with well-defined borders. The acinar cells showed intensely basophilic cytoplasm in the basal region and eosinophilic cytoplasm at the apical pole, consistent with an adequate abundance of zymogen granules, as well as rounded or oval, basally located nuclei with loose chromatin and inconspicuous nucleoli. The interacinar stroma was sparse, with no evidence of edema, fibrosis, or inflammatory infiltration, and both the blood vessels and pancreatic ducts exhibited preserved morphology.

The UM-HFD group showed mild-to-moderate histopathological alterations, with predominant involvement of the acinar compartment. The analysis identified mild-to-moderate foci of acinar hypertrophy (Figure 5A), characterized by clusters of cells with increased zymogen granule content. Acinar atrophy remained mild and appeared as focal areas of reduced acinar size (Figure 5B). The evaluation also detected changes consistent with mild acinar autophagy, manifested by cytoplasmic vesicles in isolated cells or small cell clusters (Figure 5C–E). In addition, the tissue showed slight adipocyte deposition at intra- and interlobular levels (Figure 5D,E), without histological evidence of fat necrosis. Vascular assessment revealed mild capillary dilation (Figure 5E), without fibrinoid necrosis or hemorrhage. The interstitial compartment showed mild interlobular edema, whereas ductal alterations remained scarce and of low intensity, without significant periductal inflammation (Figure 5E). The analysis did not detect acinar necrosis or fat necrosis.

The MS-CD group presented a more evident histopathological damage profile, with moderate-intensity alterations affecting multiple components of the exocrine pancreas (Figure 6A). Acinar cells showed changes consistent with moderate autophagy, characterized by more extensive cytoplasmic vacuolization organized into small cell clusters (Figure 6B). Acinar atrophy was also moderate, evidenced by a broader reduction in acinar parenchymal architecture (Figure 6C,D,F). Unlike the other groups, this condition showed foci of mild acinar necrosis, with isolated cells exhibiting characteristics of coagulative necrosis (Figure 6B,C). Vascular involvement and interstitial edema were moderate, with perivascular inflammation, focal fibrin exudation, and inter- and intralobular edema (Figure 6E). Moderate ductal alterations were also observed, including ductal dilation and periductal inflammation (Figure 6B). No areas of fat necrosis were identified.

The MS-HFD group exhibited the greatest degree of overall histopathological alteration, with predominantly moderate acinar involvement. Adipocyte deposits were observed both within and between lobules (Figure 7A). Processes compatible with acinar autophagy were more extensive than in the other groups, with clusters of cells exhibiting medium to large cytoplasmic vacuoles (Figure 7B). Acinar atrophy was the most prominent alteration, with significant structural loss of the exocrine parenchyma, foci of acinar necrosis (Figure 7C), and moderate interstitial edema and inflammation (Figure 7D). Acinar hypertrophy was mild to moderate. Vascular involvement was moderate, with foci of fibrin exudation (Figure 7E). Ductal changes were moderate, characterized by ductal dilation (Figure 7F) and mild periductal inflammation. Fat necrosis was not observed.

Accordingly, the semi-quantitative analysis (Table 4) shows a severity gradient dependent on the experimental condition, with a predominance of moderate alterations in MS-HFD, moderate involvement in MS-CD, and predominantly mild alterations, with moderate components, in UM-HFD. At the same time, the control group maintains an absence of histopathological alterations, confirming the preservation of the baseline pattern.

### 3.4. Maternal Separation and a High-Fat Diet Modulate Exocrine Pancreatic Apoptosis Through an Early Context-Dependent Interactive Effect

The UM-CD group exhibited the lowest TUNEL-positive cell count per field, serving as the reference point among the experimental groups. In comparison, apoptosis increased in the MS-CD group (+246.6% vs. UM-CD), while smaller but comparable increases were seen in the UM-HFD (+82.2% vs. UM-CD) and MS-HFD (+100.9% vs. UM-CD) groups. With both factors present, the MS-HFD group showed lower TUNEL-positive cell counts than the MS-CD group; however, this difference did not reach statistical significance, suggesting that HFD did not significantly alter the degree of apoptosis in MS-treated animals.

In accordance with these findings, a two-way ANOVA revealed that MS accounted for the largest proportion of the total variance in the number of TUNEL-positive cells (47.07%), followed by the MS × diet interaction (35.84%). In comparison, diet had a smaller but significant effect (4.88% of the total variance). Consistently, multiple comparisons analysis showed significant differences between the UM-CD and UM-HFD groups, as well as between UM-CD and MS-CD (*p* < 0.0001), and between MS-CD and MS-HFD (*p* = 0.0016), with no significant differences observed between UM-HFD and MS-HFD (Figure 8).

## 4. Discussion

The results of the present study provide evidence that early stress, modeled by MS, and post-weaning exposure to an HFD induce structural, cellular, and functional alterations in the exocrine pancreas of male C57BL/6 mice, with a predominant involvement of acinar cells. These alterations manifest differentially depending on the parameter evaluated, involving changes in relative pancreatic mass, histological integrity of acinar tissue, and cell apoptosis, even in the absence of severe or necrotizing pancreatic damage. Consistent with these morphostructural findings, functional assessment revealed modulation of pancreatic enzyme activity, characterized by a trend toward increased plasma amylase activity in the MS-treated groups. However, this did not reach statistical significance, whereas plasma lipase activity increased significantly and was independently associated with both MS and HFD consumption. Based on the above, these results suggest that early stress-induced programming and subsequent nutritional challenges contribute in a complementary manner to the progressive compromise of the pancreatic exocrine compartment, affecting not only its structure but also its functional capacity.

Macroscopic analysis showed that body mass increased significantly with HFD consumption, with no evidence of MS effects or an interaction between the two factors. In contrast, absolute pancreatic mass did not differ significantly among groups. Consequently, the reduction observed in the pancreas-to-body mass ratio associated with HFD reflects disproportionate somatic growth that is not accompanied by a corresponding expansion of pancreatic tissue, indicating an alteration in the organ–body relationship rather than a decrease in pancreatic mass per se [35]. This pattern is consistent with previous studies reporting HFD-induced increases in body mass [36,37,38] without concomitant changes in absolute pancreatic mass [39], reinforcing the dominant role of dietary factors identified in the statistical analysis.

About chronic stress, previous studies have shown that various experimental models, including acoustic stress and food restriction paradigms in rats, are associated with alterations in food intake, reductions in body mass, and changes in energy expenditure patterns [40,41]. In some of these models, particularly those based on prolonged immobilization, a significant decrease in absolute pancreatic mass has also been reported [20], suggesting a possible direct effect of sustained stress on the organ’s structure. However, this effect was not observed in the present study, in which MS, a model of early chronic stress, did not significantly affect the pancreas-to-mouse mass ratio. These discrepancies likely arise because experimental stress models differ not only in the intensity and duration of the stressor but also in the developmental stage at which exposure occurs, factors that shape the nature of their biological effects. In this context, it is important to distinguish the MS paradigm from other experimental models of induced stress, as they differ both in the timing of application and in the biological processes they replicate. While the stress models with which our results are compared—such as prolonged immobilization, acoustic stress, and food restriction—are generally applied to adult animals or after early development is complete to assess the effects of stress on already mature tissues [20,40,41], MS constitutes a model of early chronic stress applied during a critical window of development (PND2–17), a period in which various physiological systems are still maturing [42]. Consequently, their effects are interpreted primarily as the result of developmental programming mechanisms that generate persistent changes in tissue structure and function, rather than as immediate physiological responses to stress [43]. This distinction is fundamental to interpreting the findings of the present study and prevents the direct extrapolation of effects observed in other stress paradigms applied at later stages of development.

This characteristic of the MS model is particularly relevant when considering pancreatic development during the early postnatal period. At this stage, the organ undergoes intense structural and functional maturation. After birth, the exocrine compartment undergoes significant morphological changes, characterized by the progressive differentiation and enlargement of acinar cells during the first weeks of life—a process that constitutes a central component of pancreatic functional maturation [44,45,46]. Simultaneously, the secretory capacity of digestive enzymes increases, and the endocrine compartment reaches a functionally consolidated state around postnatal day 28 (PND28). Therefore, exposure to stress between PND2 and PND17 likely does not result in a decrease in total pancreatic mass, but rather in functional programming of the tissue capable of modifying its structural organization and influencing its response to subsequent metabolic challenges, such as exposure to an HFD.

In line with the macroscopic changes described above, histopathological analysis was used as a complementary approach to characterize the extent of pancreatic tissue injury, as it is widely employed in experimental studies to identify characteristic histological patterns and to facilitate comparisons between experimental conditions [32,33]. However, when based on semi-quantitative ordinal scoring systems, this approach has inherent limitations, as it relies on observer-dependent categorical assessments with limited sensitivity to detect subtle but potentially biologically relevant changes. Therefore, findings from histopathological evaluation should be interpreted with caution, as subtle quantitative differences may not be fully detected using this approach and are more accurately captured through objective morphometric analyses.

Under this interpretative framework, histopathological evaluation of the exocrine pancreas revealed a pattern of damage that depended on the experimental condition, with predominant acinar involvement and an increase in injury severity associated with both dietary exposure and early stress. The UM-CD group showed no structural alterations, confirming the morphological integrity of pancreatic tissue under baseline conditions and supporting its use as a histological reference. This reference group then enabled assigning the observed changes in the other groups to the experimental factors under study. Taken together, these results suggest a differential response of the acinar compartment to the experimental factors evaluated, reflected in the variability observed between conditions.

The UM-HFD group showed structural alterations characterized by mild-to-moderate hypertrophy and features consistent with acinar cell autophagy (cytoplasmic vacuolization). These histological findings are consistent with activation of cellular stress responses aimed at maintaining homeostasis under conditions of lipid overload [47]. Simultaneously, focal areas of mild acinar atrophy appeared, suggesting heterogeneous structural remodeling within pancreatic tissue under sustained lipid overload. Previous reports have described the coexistence of acinar hypertrophy and atrophy as a morphological pattern of acinar compartment stress in experimental models, including caerulein-induced pancreatitis [48]. In line with these findings, previous studies have shown that HFD promotes intraparenchymal fat accumulation and structural alterations in the acinar compartment, associated with increased pancreatic damage scores even in the absence of overt necrosis [21,49]. In this context, the absence of acinar necrosis and significant vascular damage in the present model suggests that, despite dietary exposure, the pancreas retains mechanisms compatible with an early response to metabolic stress, which could help maintain homeostasis and limit progression to more advanced stages of damage.

In contrast, the MS-CD group showed structural alterations of the exocrine pancreas characterized by moderate acinar atrophy as the predominant finding, with a substantial reduction in the acinar component in multiple lobes. Concomitantly, changes compatible with the activation of cellular stress mechanisms were observed, including cytoplasmic vacuolization suggestive of moderate autophagy and mild acinar necrosis. This profile is consistent with evidence indicating that early stress, such as MS, induces persistent programming of the stress–immunity–metabolism axis, increasing tissue vulnerability and susceptibility to exacerbated inflammatory responses to subsequent stimuli, even in the absence of additional dietary challenge [50,51]. In line with the apoptosis analyses, the MS-CD group showed the greatest increase in TUNEL-positive cells, indicating that apoptosis constitutes a relevant pathway of cell loss in this experimental context. This finding is consistent with previous descriptions showing that different models of pancreatic injury exhibit a predominance of apoptotic cell death over necrosis under conditions of limited damage, reflecting distinct cellular response mechanisms to stress [52,53,54]. The predominance of apoptosis can also be understood in light of mechanistic models in which autophagy dysfunction acts as a critical transition event, promoting the activation of regulated cell death pathways and the initiation of limited acinar necrosis, without requiring initial massive necrosis [55,56,57]. Furthermore, the presence of mild to moderate interstitial edema, along with signs of microvascular alteration and perivascular inflammation, reinforces the interpretation of a stress-induced state of tissue alteration, without implying a defined pancreatic inflammatory process or progression to severe injury. This profile is consistent with reports that recognize limited necrosis as a threshold towards more severe forms of pancreatic damage and, at the same time, suggests that early stress-induced programming contributes to greater tissue vulnerability and susceptibility to exacerbated inflammatory responses, even in the absence of an additional dietary challenge [58].

The MS-HFD group shows the most severe overall histopathological changes, with evidence of processes compatible with marked acinar autophagy, as well as edema, interstitial inflammation, and severe acinar atrophy. This morphological pattern suggests that combined exposure to early-life stress and a HFD induces progressive, sustained tissue remodeling, reflecting a more advanced degree of damage, although not necessarily acute. Consistent with this, previous studies have shown that HFD, in combination with various chronic stressors, exerts synergistic effects that exacerbate pancreatic metabolic dysfunction, promote local lipotoxicity, and lead to a progressive loss of pancreatic parenchyma [10,59,60]. However, other studies have reported modulatory effects of HFD under chronic social stress, particularly at systemic levels of metabolism and behavior. In this regard, it has been reported that exposure to an HFD during stress can attenuate metabolic dysregulation by acting as a “comfort food” that reduces the metabolic demands associated with activation of the stress axis [61], as well as conferring selective protection against certain behavioral sequelae resulting from unpredictable chronic social stressors [62]. Taken together, these findings suggest that the effects of a HFD in stressful conditions depend critically on the type of stressor and the timing of exposure. While in models of chronic social stress during later stages of development, the HFD may modulate metabolic or behavioral responses, in the context of early stress due to MS—a critical stage of high plasticity—such exposure appears to contribute to adverse programming of the exocrine pancreas, being associated with greater histopathological damage and a progressive process of tissue remodeling.

The histopathological findings observed in the various experimental groups suggest that structural alterations in the exocrine pancreas reflect a complex tissue response with potential functional implications rather than isolated morphological changes. Acinar hypertrophy observed in experimental models has been described as an early adaptive response that increases the biosynthetic and secretory capacity of the exocrine pancreas to meet increased functional demands [44]. However, when these adaptations are accompanied by sustained disruption of autophagy, acinar cellular homeostasis is disrupted, leading to the abnormal accumulation of zymogen granules, intracellular stress, and the activation of cell death pathways, including apoptosis and necrosis, particularly in advanced stages [47,63]. In this scenario, the progressive loss of viable acinar cells can manifest morphologically as areas of acinar atrophy and, functionally, as a reduction in effective exocrine mass, even in the absence of extensive necrosis [44]. In addition, ductal abnormalities assume functional significance, as the pancreatic ductal epithelium regulates the volume and composition of pancreatic juice by secreting bicarbonate and water. Structural changes in the ductal compartment have been associated with decreased ductal flow and bicarbonate secretion, promoting intrapancreatic stasis and creating a microenvironment conducive to the premature activation of zymogens [64,65]. Given the above, this histopathological profile is consistent with a comprehensive involvement of the acino-ductal unit, characterized by the coexistence of initially adaptive responses and processes of tissue damage and remodeling, which do not necessarily immediately result in established exocrine insufficiency but could modulate pancreatic secretory function in the early or intermediate stages of the process.

In line with this interpretation, the assessment of plasma amylase and lipase activity provided further insight into the physiological consequences of the observed histopathological changes. Since acinar cells are the primary source of these digestive enzymes, measuring their activity aimed to determine whether the structural alterations induced by MS and HFD translated into changes in pancreatic exocrine function. Although plasma amylase activity showed only a trend toward increased activity in the MS groups compared with unstressed controls, the MS factor accounted for a substantial proportion of the total variance (15.9%), suggesting a biologically meaningful contribution despite the lack of statistical significance.

The lack of statistically significant differences in plasma amylase activity should therefore be interpreted with caution. Given the relatively small sample size used for the biochemical analyses and the inherent high interindividual variability, the study may not have had sufficient statistical power to detect small-to-moderate effects. Under these conditions, the presence of a consistent trend together with a substantial proportion of explained variance is compatible with a type II error rather than the absence of a true biological effect. In addition, the timing of the enzymatic assessment may have further limited the detection of robust changes. Plasma samples were collected at the end of the 16-week dietary intervention period, several weeks after the cessation of the early stress protocol (PND2–PND17). In experimental models, circulating pancreatic enzyme activity often represents a transient response to stress or inflammatory stimuli, with early peaks followed by partial or complete normalization over time. For example, in studies of acute pancreatitis induction, where amylase and lipase activity are assessed before the stimulus and at 4, 8, and 24 h afterward, a maximum increase has been described within the first few hours, followed by a gradual return to baseline levels around 24 h [66,67]. Similarly, in models of chronic immobilization stress, measurements taken immediately after the cessation of the stressor (minutes later) have detected acute changes in enzyme activity that do not necessarily persist in later assessments [20]. As a result, late-stage measurements may underestimate earlier functional alterations induced by early-life stress.

Unlike plasma amylase activity, plasma lipase activity increased significantly, particularly in the MS-HFD group, suggesting greater sensitivity of this component of pancreatic exocrine function to early stress and subsequent dietary challenges. This pattern is consistent with the differential adaptation described for digestive enzymes in experimental models, in which lipid overload is associated with a preferential increase in lipase activity, even when other enzyme markers show limited or transient changes [68,69]. Consequently, statistical analysis revealed significant and independent effects of MS and HFD, with no synergistic interaction between the two factors, indicating a cumulative modulation of lipase activity. Taken together, these findings suggest that, even considering the methodological and temporal limitations of the sampling, the higher plasma lipase activity observed likely reflects both a sustained dietary adaptation to excess lipids and greater functional vulnerability of the acinar tissue previously conditioned by early stress.

Although the present findings support the notion that early-life stress may increase the structural vulnerability of the exocrine pancreas to later metabolic challenges, their translational interpretation should be considered with caution. The study was conducted exclusively in male mice, using a model of severe MS combined with early weaning and a single post-weaning dietary challenge. Moreover, functional assessments of exocrine pancreatic output were limited, precluding direct conclusions regarding digestive capacity or disease progression. Therefore, the present results should be interpreted as preliminary morphological evidence of increased susceptibility, rather than as a direct model of human pancreatic disease.

This study has several limitations that should be considered when interpreting the results. First, the analysis was restricted to morphometric, histopathological, and functional assessments, without including specific molecular markers that would have enabled a deeper mechanistic characterization of the pathways underlying the observed exocrine pancreatic alterations. In particular, the lack of evaluation of endoplasmic reticulum stress and adaptive unfolded protein response markers (such as GRP78 and the spliced form of X-box binding protein 1, XBP1s), autophagy-related proteins (including LC3B), markers of acinar cell identity and plasticity (such as the transcription factor SOX9), as well as inflammatory mediators (e.g., TNF-α and IL-6), limits the ability to determine whether the structural and functional changes reflect adaptive responses, disrupted cellular homeostasis, or early remodeling processes within the acinar compartment. Second, the study was conducted exclusively in male mice, which limits the generalizability of the findings to females. Accumulating evidence indicates that sex differences crucially influence physiological and pathological responses to early-life stress [70,71] and dietary challenges [72,73]. Female rodents exhibit distinct hypothalamic–pituitary–adrenal axis reactivity, inflammatory profiles, and lipid handling compared to males, which may result in differential vulnerability or adaptive capacity of the exocrine pancreas following MS and HFD. Therefore, the exclusive use of male mice may have masked sex-specific protective or exacerbating mechanisms, underscoring the need for future studies incorporating both sexes to elucidate the interplay between early-life stress, diet, and sex-dependent pancreatic responses.

Furthermore, the experimental design does not permit a precise determination of the reversibility of the observed alterations or the establishment of direct causal relationships between histological and functional changes and metabolic outcomes, given the cross-sectional nature of the analysis. Finally, although MS is a widely validated model of early-life stress, its effects may vary with duration, the timing of application, and concomitant environmental conditions, which could contribute to heterogeneity in results and should be considered when comparing these findings with those from other stress paradigms. Finally, a key limitation of this study is the small sample size (*n* = 5 per group), which reduces statistical power to detect small to moderate effects, particularly in two-way ANOVA analyses of interactions between early-life stress and diet. As a result, non-significant or negative findings require cautious interpretation, since limited power may explain the absence of statistical significance rather than a true lack of effect. In addition, including only one male offspring per litter means the litter, rather than the individual animal, serves as the effective experimental unit, introducing a risk of pseudoreplication. Together, these design constraints reduce the robustness of some comparisons and should be considered when interpreting effect sizes, especially for interaction effects.

## 5. Conclusions

Maternal separation and post-weaning high-fat diet exposure induced distinct but complementary alterations in the exocrine pancreas of adult mice. While the high-fat diet primarily affected body mass and pancreatic enzyme activity, early-life stress was more strongly associated with apoptosis and histopathological alterations involving the acinar, vascular, and ductal compartments. Animals exposed to both factors exhibited the most pronounced structural alterations, indicating that early adverse experiences modify the exocrine pancreatic response to subsequent metabolic challenges.

These findings suggest that maternal separation does not necessarily cause overt exocrine pancreatic injury by itself but may program increased structural and functional vulnerability that becomes evident under later nutritional stress. The study expands current knowledge regarding the long-term consequences of adverse early-life experiences beyond the endocrine pancreas. It highlights the exocrine pancreas as a previously underexplored target of developmental programming. Further studies incorporating quantitative morphometric and molecular approaches are needed to elucidate the mechanisms underlying this increased susceptibility.

## Figures and Tables

**Figure 1:**
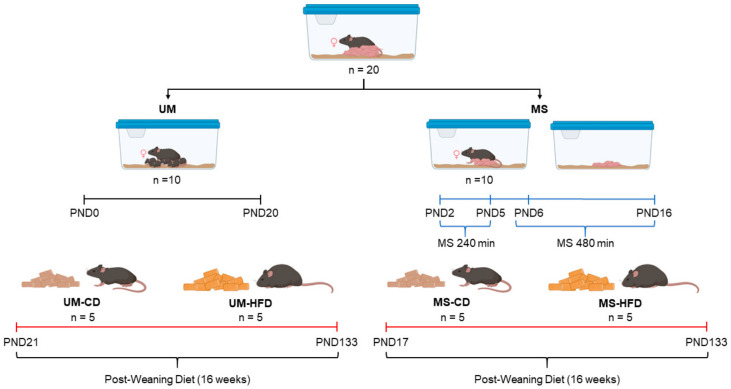
Overview of the experimental design and study timeline. Male C57BL/6 mice were assigned to either the unmanipulated (UM) or maternal separation (MS) groups during the early postnatal period. Following weaning (PND17 for MS and PND21 for UM), animals were fed either a control diet (CD) or a high-fat diet (HFD) for 16 weeks, generating four experimental groups: UM-CD, UM-HFD, MS-CD, and MS-HFD. The following outcomes were evaluated: body mass, pancreatic mass, plasma amylase and lipase activities, histopathological evaluation of the exocrine pancreas, and apoptosis. Abbreviations: PND, postnatal day. Created with BioRender.com.

**Figure 2:**
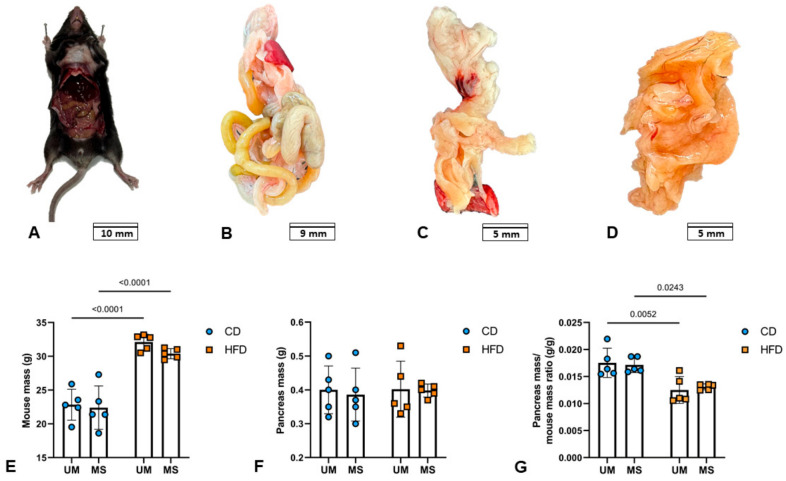
Macroscopic identification and dissection of the pancreas and quantitative analysis of body and pancreatic mass. (**A**–**D**) Representative images of the pancreatic dissection procedure. (**E**) Body mass. (**F**) Pancreatic mass. (**G**) Pancreas-to-body mass ratio in male C57BL/6 mice from the unmanipulated (UM) or maternal separation (MS) groups and fed either a control diet (CD) or a high-fat diet (HFD). Data are presented as mean ± SD. Each dot represents one animal (*n* = 5 per group). Statistical significance was assessed by two-way ANOVA followed by Tukey’s multiple-comparisons test. *p* < 0.05.

**Figure 3:**
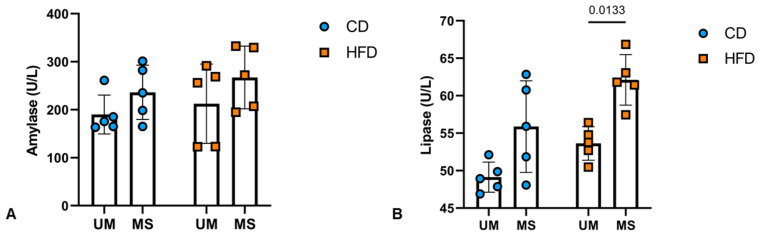
Plasma amylase and lipase activities in male C57BL/6 mice from the unmanipulated (UM) or maternal separation (MS) groups and fed either a control diet (CD) or a high-fat diet (HFD). (**A**) Plasma amylase activity. (**B**) Plasma lipase activity. Data are presented as mean ± SD. Each dot represents one animal (*n* = 5 per group). Statistical significance was assessed by two-way ANOVA followed by Tukey’s multiple-comparisons test. *p* < 0.05.

**Figure 4:**
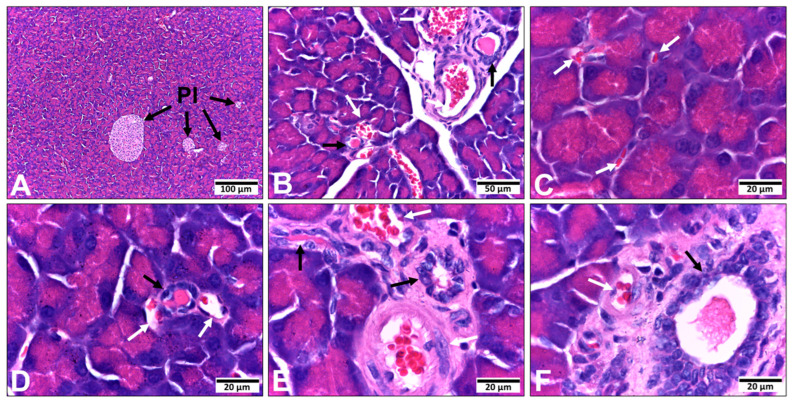
Representative histological sections of the exocrine pancreas from male C57BL/6 mice in the unmanipulated control diet group (UM-CD). (**A**) Overview of the pancreas showing preserved exocrine architecture and pancreatic islets (PI). (**B**) Normal organization of the exocrine parenchyma with pancreatic ducts (black arrows) and blood vessels (white arrows). (**C**) Acinar cells with preserved morphology, characterized by basophilic basal cytoplasm and apical eosinophilic zymogen granules. (**D**) Intercalated duct (black arrow) and adjacent microvasculature (white arrows). (**E**) Intralobular duct (black arrow) associated with blood vessels (white arrows). (**F**) Interlobular duct (black arrow) and adjacent blood vessels (white arrows) within the connective tissue. H&E staining. Scale bars as indicated.

**Figure 5:**
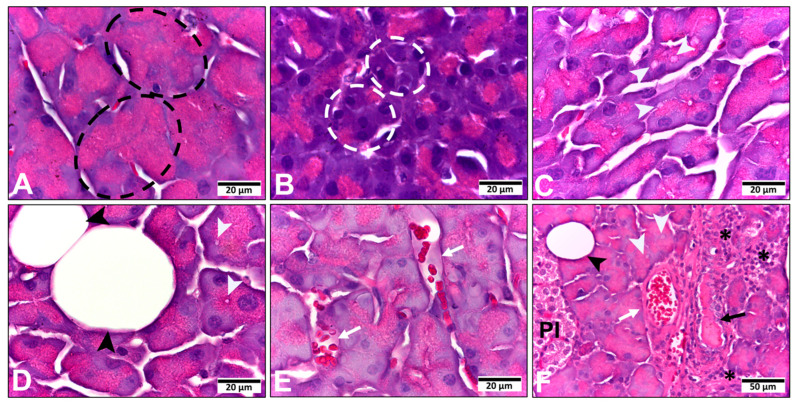
Representative histological sections of the exocrine pancreas from male C57BL/6 mice in the unmanipulated high-fat diet group (UM-HFD). (**A**) Foci of acinar hypertrophy (dashed circles) are characterized by increased cell size and accumulation of zymogen granules. (**B**) Areas of acinar atrophy (dashed circles) with reduced acinar size and cytoplasmic volume. (**C**) Acinar cells containing cytoplasmic vesicles (arrowheads), consistent with autophagic vacuolization. (**D**) Intralobular adipocytes (black arrowheads) are associated with acinar cells showing cytoplasmic vesicles (white arrowheads). (**E**) Capillary dilation (white arrows) and mild interstitial alterations within the exocrine parenchyma. (**F**) Pancreatic islet (PI) adjacent to exocrine tissue, with an intralobular duct (black arrow), blood vessels (white arrows), and focal inflammatory infiltrate (asterisk). H&E staining. Scale bars as indicated.

**Figure 6:**
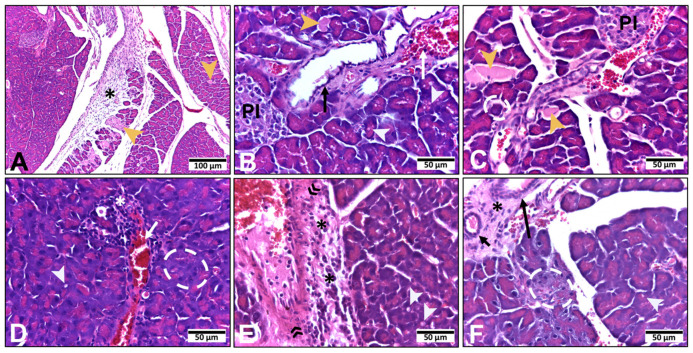
Representative histological sections of the exocrine pancreas from male C57BL/6 mice in the maternal separation control diet (MS-CD) group. (**A**) Interlobular region showing inflammatory infiltrate (asterisk) and expansion of the interlobular connective tissue. (**B**) Dilated intralobular duct (black arrow) adjacent to a pancreatic islet (PI), with acinar cells containing cytoplasmic vesicles (white arrowheads) and necrotic cells (yellow arrowheads); the white arrow indicates a blood vessel. (**C**) Foci of acinar necrosis (yellow arrowheads) associated with acinar atrophy (dashed circle). (**D**) Inflammatory infiltrate (asterisk), vascular dilation (white arrow), and acinar atrophy (dashed circle). (**E**) Expanded interlobular connective tissue showing perivascular inflammation (asterisks), interstitial edema, and fibrin exudation (double arrowhead). (**F**) Acinar atrophy (dashed circle) associated with periductal inflammatory infiltrate (asterisk). Hematoxylin and eosin (H&E) staining. Scale bars as indicated.

**Figure 7:**
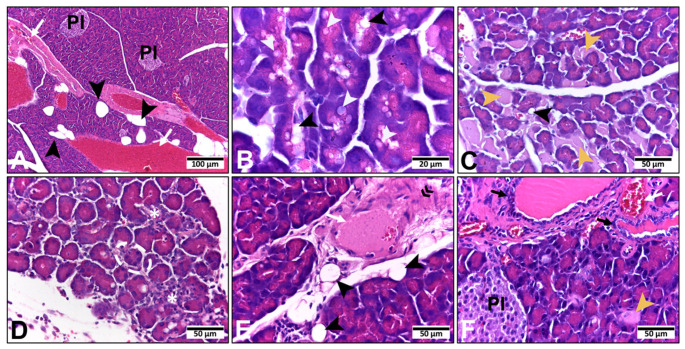
Representative histological sections of the exocrine pancreas from male C57BL/6 mice in the maternal separation high-fat diet group (MS-HFD). (**A**) Intra- and interlobular adipocytes (black arrowheads) are associated with the pancreatic parenchyma. (**B**) Acinar cells containing cytoplasmic vesicles (white arrowheads) and lipid droplets (black arrowheads). (**C**) Acinar atrophy associated with foci of acinar necrosis (yellow arrowheads). (**D**) Exocrine parenchyma showing interstitial inflammatory infiltrate (asterisk), edema and acinar atrophy (dashed circle). (**E**) Vascular alterations with fibrin exudation (double arrowhead). (**F**) Dilated intralobular duct (black arrow). H&E staining. Scale bars as indicated.

**Figure 8:**
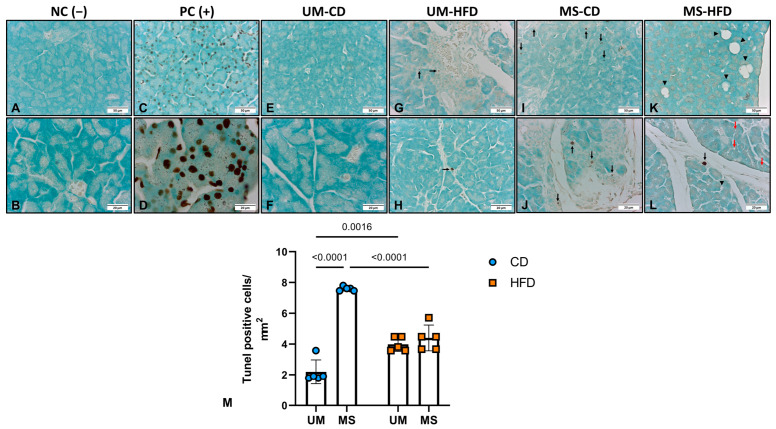
Representative TUNEL-stained sections of the exocrine pancreas and quantitative analysis of apoptosis in male C57BL/6 mice from the unmanipulated (UM) or maternal separation (MS) groups fed either a control diet (CD) or a high-fat diet (HFD). (**A**,**B**) Negative controls showing the absence of TUNEL staining. (**C**,**D**) Positive controls confirming the specificity of the TUNEL assay. (**E**,**F**) UM-CD group. (**G**,**H**) UM-HFD group. (**I**,**J**) MS-CD group. (**K**,**L**) MS-HFD group. Black arrows indicate TUNEL-positive nuclei, arrowheads indicate adipocytes, and red arrows indicate lipid droplets. (**M**) Quantification of TUNEL-positive nuclei (cells/mm^2^). Data are presented as mean ± SD. Each dot represents one animal (*n* = 5 per group). Statistical significance was assessed using two-way ANOVA followed by Tukey’s multiple comparisons test. *p* < 0.05.

**Table 1:** Diets were administered according to the experimental group: control diet (CD) and high-fat diet (HFD). The vitamin and mineral mixtures followed the American Institute of Nutrition’s AIN-93G standard for rodents. The high-fat diet provided 49% of its energy from fat.

Ingredients	CD	HFD
Casein (>85% protein)	200.0	230.0
L-cystine (g/kg)	3.0	3.0
Cornstarch (g/kg)	529.486	299.472
Sucrose (g/kg)	100.0	100.0
Soybean oil (g/kg)	70.0	70.0
Lard (g/kg)	-	200.0
Fiber (g/kg)	50.0	50.0
Vitamin mixture (g/kg)	10.0	10.0
Mineral mixture (g/kg)	35.0	35.0
Choline bitartrate (g/kg)	2.5	2.5
Antioxidant (g/kg)	0.014	0.028
Total (g)	1000.0	1000.0
Energy (kcal/g)	3.95	4.95
Carbohydrate (% Energy)	64.0	32.0
Protein (% Energy)	19.0	19.0
Lipid (% Energy)	17.0	49.0

**Table 2:** Semi-quantitative scoring system used to evaluate exocrine pancreatic injury. Histopathological alterations were graded from 0 (absence of lesion) to 3 (severe lesion) according to criteria adapted from Zhang and Rouse [32] and supplemented with complementary morphological descriptions based on Rouse et al. [33].

Score	Acinar Hypertrophy	Acinar Autophagy	Acinar Necrosis	Vascular Injury	Interstitial Edema, Inflammation, and Hemorrhage	Fat Necrosis	Ductal Alterations	Acinar Atrophy
0	No increase in acinar cell size	No morphological evidence of autophagy	No necrotic acinar cells	No vascular alterations	No edema, inflammation, or hemorrhage	No fat necrosis	No ductal dilatation, inflammation, or fibrosis	No acinar atrophy
1	Isolated acinar cells with increased apical cytoplasmic volume (zymogen granule region)	Isolated acinar cells with small cytoplasmic vacuoles	Isolated acinar cells with coagulative necrosis, loss of zymogen granules, and cytoplasmic eosinophilia	Capillary dilatation and perivascular leukocyte migration in capillaries, venules, or small veins	Mild interlobular edema without inflammation or hemorrhage	Small foci of fat necrosis with basophilic deposits and neutrophilic infiltration	Mild ductal alterations characterized by centroacinar cell proliferation, intercalated duct hyperplasia, and mild intralobular inflammation	Acinar atrophy affecting <25% of lobules, with minimal fat infiltration
2	Clusters of acinar cells with nodular hypertrophy	Small clusters of acinar cells with medium or large cytoplasmic vacuoles, some containing zymogen granules or amorphous material	Clusters of necrotic acinar cells with cellular debris, neutrophilic infiltration, and occasional hemorrhage	Fibrinoid necrosis of arterioles or small arteries with fibrin exudation and perivascular inflammation	Moderate intralobular edema with inflammation and hemorrhage	Large areas of fat necrosis with prominent inflammatory infiltrate	Moderate ductal alterations characterized by intralobular duct dilatation, periductal inflammation, and early fibrosis	Acinar atrophy affecting 25–50% of lobules, with moderate fat infiltration
3	Hypertrophy affecting most acinar cells within a lobule	Groups of acinar cells with large or giant cytoplasmic vacuoles, often coalescent	Extensive multilobular acinar necrosis with inflammatory infiltrate and hemorrhage	Fibrinoid necrosis, hemorrhage, and inflammatory infiltration involving medium-sized arteries	Severe edema with marked inflammation and hemorrhage	Extensive fat necrosis with calcification, inflammation, and/or hemorrhage	Severe ductal alterations characterized by marked duct dilatation, epithelial proliferation, periductal inflammation, and lobular fibrosis	Acinar atrophy affecting >50% of lobules, with extensive adipose tissue replacement

**Table 3:** Detailed results of the two-way ANOVA using mouse data at the end of the experiment.

Parameters	MS					Diet					Interaction				
	% Total Variation	*p* Value	F (1, 16)	η^2^p	95% CI η^2^p	% Total Variation	*p* Value	F (1, 16)	η^2^p	95% CI η^2^p	% Total Variation	*p* Value	F (1, 16)	η^2^p	95% CI η^2^p
Mouse mass	1.28	0.2680	1.317	0.076	0.0002–0.431	**82.72**	<0.0001	85.10	0.842	0.715–0.931	0.45	0.5065	0.5065	0.028	0.0001–0.350
Pancreas mass	**0.55**	0.7700	0.08843	0.0055	0.000–0.188	0.33	0.8200	0.05349	0.0033	0.000–0.162	0.17	0.8709	0.8709	0.0017	0.000–0.140
Pancreas mass/mouse mass ratio	0.03	NS	0.01237	0.0008	0.000–0.110	**61.53**	0.0001	0.2908	0.620	0.360–0.820	0.69	NS	NS	0.018	0.000–0.270
TUNEL	**51.63**	<0.0001	110.0	0.873	0.720–0.945	2.96	0.0231	6.309	0.283	0.030–0.560	37.90	<0.0001	<0.0001	0.835	0.650–0.920
Amylase	**15.90**	NS	3.198	0.167	0.000–0.410	4.44	NS	0.8926	0.053	0.000–0.300	0.11	NS	NS	0.0014	0.000–0.135
Lipase	**43.36**	0.0004	20.07	0.556	0.300–0.780	21.51	0.0061	9.960	0.384	0.110–0.630	0.57	0.6159	0.6159	0.016	0.000–0.250

The most powerful factor influencing total variance is in bold for each parameter. Whenever a factor or an interaction did not significantly influence the parameter, it is described as NS.

**Table 4:** Semi-quantitative histopathological scores of exocrine pancreatic alterations in male C57BL/6 mice subjected to maternal separation (MS) or standard rearing (UM) and fed a control diet (CD) or high-fat diet (HFD). Scores were assigned using an ordinal scale from 0 to 3, where 0 indicates absence of alterations and 1, 2, and 3 correspond to mild, moderate, and severe changes, respectively. Values are presented as median scores. UM-CD, unmanipulated control diet; UM-HFD, unmanipulated high-fat diet; MS-CD, maternal separation control diet; MS-HFD, maternal separation high-fat diet; *n* = 5 animals per group.

Categories	Median (P25–P75)			
	UM-CD	UM-HFD	MS-CD	MS-HFD
Acinar hypertrophy	0.0 (0.0–0.0)	2.0 (0.5–2.5)	1.0 (0.5–1.0)	1.0 (1.0–1.5)
Acinar autophagy	0.0 (0.0–0.0)	1.0 (0.5–2.0)	2.0 (1.0–2.5)	2.0 (2.0–2.0)
Acinar necrosis	0.0 (0.0–0.0)	0.0 (0.0–0.0)	1.0 (0.0–2.0)	1.0 (0.0–2.0)
Vascular injury	0.0 (0.0–0.0)	1.0 (0.5–1.5)	2.0 (1.0–2.5)	2.0 (1.0–2.0)
Interstitial edema, inflammation, and hemorrhage	0.0 (0.0–0.0)	1.0 (0.5–2.0)	2.0 (1.5–2.0)	2.0 (1.5–2.0)
Fat necrosis	0.0 (0.0–0.0)	0.0 (0.0–0.0)	0.0 (0.0–0.0)	0.0 (0.0–0.0)
Ductal alterations	0.0 (0.0–0.0)	0.0 (0.0–1.0)	2.0 (1.0–2.0)	2.0 (1.0–2.0)
Acinar atrophy	0.0 (0.0–0.0)	1.0 (0.5–2.0)	2.0 (1.0–2.5)	3.0 (2.0–3.0)

## Data Availability

The raw data supporting the conclusions of this article will be made available by the authors, without undue reservation.

## Abbreviations

The following abbreviations are used in this manuscript:

ACEs	Adverse childhood experiences
CD	Control diet
CEMyQ	Center of excellence in morphological and surgical studies
HFD	High-fat diets
MS	Maternal separation
MS-CD	Maternal separation group with control diet
MS-HFD	Maternal separation group with a high-fat diet
PND	Postnatal day
RT	Room temperature
UM	Unmanipulated
UM-CD	Unmanipulated group with control diet
UM-HFD	Unmanipulated group with a high-fat diet
